# Fractal correlation properties of heart rate variability as a biomarker of endurance exercise fatigue in ultramarathon runners

**DOI:** 10.14814/phy2.14956

**Published:** 2021-07-22

**Authors:** Bruce Rogers, Laurent Mourot, Gregory Doucende, Thomas Gronwald

**Affiliations:** ^1^ College of Medicine University of Central Florida Orlando FL USA; ^2^ EA3920 Prognostic Factors and Regulatory Factors of Cardiac and Vascular Pathologies Exercise Performance Health Innovation (EPHI) platform, University of Bourgogne Franche‐Comté Besançon France; ^3^ National Research Tomsk Polytechnic University Tomsk Oblast Russia; ^4^ Université de Perpignan Via Domitia Laboratoire Européen Performance Santé Altitude (LEPSA) Besançon France; ^5^ Faculty of Health Sciences Department of Performance, Neuroscience Therapy and Health MSH Medical School Hamburg University of Applied Sciences and Medical University Hamburg Germany

**Keywords:** DFA a1, endurance exercise, fatigue, marathon, running

## Abstract

Although heart rate variability (HRV) indexes have been helpful for monitoring the fatigued state while resting, little data indicate that there is comparable potential during exercise. Since an index of HRV based on fractal correlation properties, alpha 1 of detrended fluctuation analysis (DFA a1) displays overall organismic demands, alteration during exertion may provide insight into physiologic changes accompanying fatigue. Two weeks after collecting baseline demographic and gas exchange data, 11 experienced ultramarathon runners were divided into two groups. Seven runners performed a simulated ultramarathon for 6 h (Fatigue group, FG) and four runners performed daily activity over a similar period (Control group, CG). Before (Pre) and after (Post) the ultramarathon or daily activity, DFA a1, heart rate (HR), running economy (RE) and countermovement jumps (CMJ) were measured while running on a treadmill at 3 m/s. In Pre versus Post comparisons, data showed a decline with large effect size in DFA a1 post intervention only for FG (Pre: 0.71, Post: 0.32; d = 1.34), with minor differences and small effect sizes in HR (d = 0.02) and RE (d = 0.21). CG showed only minor differences with small effect sizes in DFA a1 (d = 0.19), HR (d = 0.15), and RE (d = 0.31). CMJ vertical peak force showed fatigue‐induced decreases with large effect size in FG (d = 0.82) compared to CG (d = 0.02). At the completion of an ultramarathon, DFA a1 decreased with large effect size while running at low intensity compared to pre‐race values. DFA a1 may offer an opportunity for real‐time tracking of physiologic status in terms of monitoring for fatigue and possibly as an early warning signal of systemic perturbation.

## Graphical abstract

The aim of this report is to evaluate the change in exercise associated DFA a1 dynamics toward the end of a simulated ultramarathon and compare this to changes in HR and running economy while still performing dynamic exercise. At the completion of an ultramarathon, DFA a1 decreased with large effect size while running at low intensity compared to pre‐race values. Therefore, DFA a1 may offer an opportunity for assessment and real‐time tracking of physiologic status in terms of monitoring for fatigue and possibly as an early warning signal of systemic perturbation.

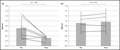

## 1 INTRODUCTION

The classification of endurance exercise fatigue encompasses diverse models and theories (Abbiss & Laursen, 2005), components (Carriker, 2017), and various aspects of muscular function (Wan et al., 2017), biochemical balance (Jastrzębski et al., 2015) as well as both the central and peripheral nervous systems (Davis & Walsh, 2010; McMorris et al., 2018; Martínez‐Navarro et al., 2019; Martin et al., 2018; for an overview see Ament & Verkerke, 2009). Objective means to quantify fatigue related to endurance exercise may include various modalities including salivary hormone markers (Deneen & Jones, 2017), muscle enzyme elevation (Martínez‐Navarro et al., 2019), blood lactate concentration (Jastrzębski et al., 2015), markers of substrate availability (Schader et al., 2020), cortical activity (Ludyga et al., 2016), functional testing such as the counter movement jump (Wu et al., 2019) and measures of running economy (Scheer et al., 2018). Fatigue can be also measured subjectively through “rating of perceived effort” (RPE, Halperin & Emanuel, 2020) such as the well‐known Borg scale (Borg, 1982).

Although well established, none of these tools are easily implemented for practical usage in the vast majority of endurance athletes. Since exercise‐related fatigue is an inevitable consequence of a long duration endurance session, an easily available objective biomarker using a low‐cost consumer wearable device would be ideal. While resting heart rate (HR) variability (HRV) may provide information on functional overreaching, and post exercise HRV may indicate autonomic recovery status (Manresa‐Rocamora et al., 2021; Stanley et al., 2013), neither modality can answer the question of whether a specific exercise endeavor is leading to a fatigued state as the activity occurs.

Recently, a nonlinear index of HRV based on fractal correlation properties termed alpha 1 (short‐term scaling exponent) of detrended fluctuation analysis (DFA a1) has been shown to change with increasing exercise intensity (Gronwald & Hoos, 2020). This index represents the fractal, self‐similar nature of cardiac beat‐to‐beat intervals. At low exercise intensity, DFA a1 values usually are near 1 or slightly above, signifying a well correlated, fractal pattern. As intensity rises, the index will drop past 0.75 near the aerobic threshold (AT) then approach uncorrelated, random patterns represented by values near 0.5 at higher work rates (Rogers, Giles, Draper, Hoos et al., 2021). The underlying mechanism for this behavior is felt to be due to alterations in autonomic nervous system balance, primarily withdrawal of the parasympathetic branch and enhancement of the sympathetic branch as well as other potential factors (Gronwald et al., 2020). As opposed to other HRV indexes that reach a nadir value at the aerobic threshold (SDNN: the total variability as the standard deviation of all normal RR intervals; SD1: standard deviation of the distances of the points from the minor axis in the Poincaré plot), DFA a1 has a wide dynamic range sufficient to differentiate mild versus moderate versus severe intensity domains. For example, at the AT, a DFA a1 near 0.75 is usually present (Rogers, Giles, Draper, Hoos et al., 2021), whereas SDNN and SD1 are already at their lowest values (Gronwald et al., 2020). One advantageous property of DFA a1 revolves around its dimensionless nature, as values appear to apply to an individual regardless of fitness status. For example, a value of 0.5 corresponds to an exercise intensity well above the AT in most individuals without having prior knowledge of the current HR or power (Gronwald et al., 2020). In addition to its recent usage to delineate the AT during exercise testing, DFA a1 has an extensive literature as a final common pathway of assessing total body “organismic demand” (Gronwald & Hoos, 2020). This concept refers to DFA a1 status as an index of overall systemic internal load rather than being purely related to isolated single factor measures of external load such as cycling power, or metrics of subsystem internal loads such as HR, respiratory rate, or VO_2_. Therefore, the dimensionless index DFA a1 shows great potential as a descriptor of the Network Physiology of Exercise (NPE), recently introduced by Balagué et al., (2020). In particular, this index is well suited for the demarcation of the complex dynamics of internal load development over the course of prolonged endurance exercise as well as for the assessment of athletes' fatigued state while still in the process of exercising.

Although various endurance exercise modalities can lead to fatigue, the ultramarathon represents one of the most extreme examples. As defined by a run distance of over 42 km with a variety of surface/terrain/elevation characteristics (Scheer et al., 2020), it has been associated with electrolyte imbalance, severe muscle damage, end organ dysfunction, altered oxygen cost of running, and hormonal dysregulation (Knechtle & Nikolaidis, 2018; Ramos‐Campo et al., 2016). At the same time, the pace is generally considered moderate, with only slight lactate elevations above baseline noted (Jastrzębski et al., 2015; Ramos‐Campo et al., 2016). Therefore, it represents an extreme setting of prolonged but moderate level exercise intensity that can lead to major systemic perturbation. Since DFA a1 has been shown to be a marker of overall organismic demand, it would be of interest to explore its behavior after such an endeavor. In addition, since it has also been noted to be a proxy for the aerobic threshold, alteration of this relationship may indicate the need for pace adjustment for the purpose of intensity distribution. Although relatively short durations of exercise below the AT do not seem to lead to major alterations in DFA a1 behavior (Rogers, 2020), physiologic disruption produced by an ultramarathon certainly could do so. Hence, the aim of this report is to evaluate the change in exercise associated DFA a1 dynamics toward the end of a simulated ultramarathon and compare this to changes in HR and running economy while still performing dynamic exercise.

## 2 MATERIALS AND METHODS

### 2.1 Participants

Eleven experienced (nine male, two female) ultramarathon runners without major past medical history, medications, or recent illness were recruited for the study. All had purposefully trained for an ultramarathon and were experienced in performing a race of greater than 50 km or longer than 6 h in total duration.

### 2.2 Baseline assessment

As part of the baseline assessment, participants performed a familiarization of countermovement jumps (CMJ) practice with an emphasis on the speed of jump. An incremental treadmill test to exhaustion was done to determine peak oxygen uptake (VO_2MAX_), the first and second ventilatory thresholds 2 weeks prior to the ultramarathon run. After a warm‐up of about 10 min at 3 m/s, the initial running speed was set at 3.6 m/s with the first stage lasting 2 min. The speed was then progressively increased by 0.28 m/s every 2 min until exhaustion. Breath‐by‐breath gas exchange was continuously measured via metabolic cart (Metalyzer 3B‐R3system; Cortex Biophysics, Leipzig, Germany). Ventilatory thresholds were determined visually with the first threshold defined by the V slope method and second threshold by the change in VCO_2_/ventilation ratio (Beaver et al., 1986). VO_2MAX_ was defined as the average VO_2_ over the last 60 s of the test. Peak effort was confirmed by failure of VO_2_ and/or HR to increase with further increases in work rate. Pertinent demographic data are shown in Table 1 including age, height, weight, years of training, weekly training volume, and results of the gas exchange testing. Participants did not consume caffeine, alcohol, or any stimulant for the 24 h before testing. The experimental design of the study was approved by the local Human Research Ethic Committee (2016‐A00511‐50), conducted in conformity with the latest version of the Declaration of Helsinki and written informed consent for all participants was obtained.

**TABLE 1:** Demographic data and data from the baseline assessment of all participants (*n* = 11)

Group	Age	Sex	BW [Kg]	Ht [cm]	Yrs training	Hrs/wk training	VO_2MAX_ [ml/kg/min]	VT1 [ml/kg/min]	VT2 [ml/kg/min]
FG 1	20	M	70	190	6	13	80	52	68
FG 2	24	M	65	175	10	12	75	48	65
FG 3	22	M	81	186	10	11	74	47	63
FG 4	44	F	54	162	6	11	63	39	52
FG 5	45	M	64	170	5	5	55	36	45
FG 6	43	M	72	176	30	5	53	35	43
FG 7	49	M	71	170	12	8	52	34	42
Mean±SD	35 (±12)	–	68 (±8)	176 (±9)	11 (±8)	9 (±3)	64 (±11)	42 (±7)	54 (±10)
CG 1	24	M	67	162	8	15	75	46	62
CG 2	32	M	68	178	6	9	75	47	65
CG 3	40	M	68	177	20	9	70	45	60
CG 4	42	F	60	168	3	4	49	30	41
Mean ± SD	35 (±7)	–	66 (±3)	171 (±7)	9 (±6)	9 (±4)	67 (±11)	42 (±7)	57 (±9)
d	0.07	–	0.33	0.48	0.25	0.01	0.22	0.06	0.27

Group: Fatigue group with number of the participant (FG) and Control group with number of the participant (CG), Age, current age, Sex; BW, Body weight; Ht, Height; Yrs training, total years of marathon training; Hrs/wk training, approximate hours per week of marathon‐related training; VO_2MAX_, peak oxygen uptake reached on baseline ramp test; VT1, first ventilatory threshold; VT2, second ventilatory threshold. Mean (± standard deviation, SD) and Cohen's d for group comparisons in last row.

### 2.3 Study protocol

Initially, all participants underwent a CMJ testing sessions with 3 CMJ trials and 30 s rest between to assess fatigue‐induced changes in the neuromuscular function (Claudino et al., 2017). The maximum jump height and the vertical peak force normalized per the participants’ body mass(N/kg) were measured using a portable force platform (Quattro‐Jump, Kistler, Winterthur, Switzerland) at a sampling rate of 500 Hz. The average values of the 3 CMJ trials were used in the subsequent statistical analysis. All participants then performed a treadmill run (Pre) at a fixed velocity of 3 m/s for a duration of 5 min the day before the simulated ultramarathon for measurements of oxygen uptake (VO_2_). Breath‐by‐breath gas exchange was continuously measured by the same metabolic cart as in the initial assessment (Metalyzer 3B‐R3 system; Cortex Biophysics, Leipzig, Germany). VO_2_ was averaged over the last 1 min to estimate the running economy (Bontemps et al., 2020). The following day, seven participants ran a simulated ultramarathon for approximately 6 h (Fatigue group, FG, see Table 2), while the remaining four participants (Control group, CG) did normal nonstrenuous daily activity for 6 h. Participants ran on an 11.5‐km off road trail loop at a freely chosen pace (with an elevation change of 550 m) without rest periods and were allowed to ingest food and water freely. Immediately following the completion of the 6 h run or 6 h nonstrenuous activity, an identical CMJ assessment and treadmill test (Post) was performed on each individual for the same measurement parameters. No change in protocol occurred between pre and post intervention testing. Estimated running speed was calculated based on total covered distance and elapsed time.

**TABLE 2:** Pre and Post intervention data for both groups and all participants (n=11)

Group	Pre						Post						Ultramarathon		
	HR [bpm]	DFA a1	RE [ml/kg/min]	CMJ vertical peak force [N/kg]	CMJ jump height [cm]	VO_2_ run/VT1 [%]	HR [bpm]	DFA a1	RE [ml/kg/min]	CMJ vertical peak force [N/kg]	CMJ jump height [cm]	VO_2_ run/VT1 [%]	Time [h:min]	Distance [Km]	Speed [m/s]
FG 1	158	1.286	39	–	–	75%	170	0.353	41	–	–	78%	5:50	42	2.0
FG 2	125	1.192	37	21.7	32.2	77%	133	0.396	36	21.7	30.1	75%	6:35	48	2.0
FG 3	134	0.776	29	18.9	34.6	61%	134	0.356	33	17.1	29.0	70%	6:35	48	2.0
FG 4	132	0.269	36	21.6	21.7	92%	131	0.358	35	20.0	19.9	90%	5:52	44	2.1
FG 5	149	0.706	37	21.9	23.1	102%	141	0.314	37	18.4	20.5	102%	5:54	39	1.8
FG 6	141	0.313	41	20.7	26.9	117%	135	0.124	35	18.8	24.5	100%	6:15	43	1.9
FG 7	148	0.436	35	16.7	15.0	102%	143	0.317	32	16.5	13.8	93%	6:10	45	2.0
Mean±SD	141 (±11)	0.71 (±0.41)	36 (±4)	20.2 (±1.9)	25.6 (±6.6)	89 (±19)	141 (±13)	0.32 (±0.09)	36 (±3)	18.8 (±1.7)	23.0 (±5.6)	87 (±12)	6:10 (±0:19)	44 (±3)	2.0 (±0.1)
CG 1	129	1.201	34	25.7	33.7	74%	127	1.301	33	26.1	34.8	72%			
CG 2	140	0.853	36	20.2	24.5	76%	136	0.806	35	19.4	24.4	74%			
CG 3	110	1.063	32	22.4	23.7	71%	103	1.157	32	22.5	24.0	71%			
CG 4	163	0.559	38	17.9	12.3	125%	158	0.598	37	18.5	13.9	122%			
Mean±SD	135 (±22)	0.92 (±0.28)	35 (±3)	21.6 (±3.3)	23.6 (±8.8)	87 (±25)	131 (±22)	0.97 (±0.32)	34 (±2)	21.6 (±3.4)	24.3 (±8.5)	85 (±21)			
d	0.34	0.56	0.38	0.53	0.28	0.12	0.58	3.25	0.49	1.17	0.19	0.12			

Group, Fatigue group with number of the participant (FG) and Control group with number of the participant (CG); HR, average heart rate; DFA a1, short‐term scaling exponent alpha1 of detrended fluctuation analysis; RE, running economy via oxygen uptake; CMJ, counter movement jump assessment (please consider that there is one data pair missing in FG due to technical issues) ; VO_2_ run/VT1, ratio of the oxygen uptake measured during the Pre or Post 3 m/s treadmill run to that of the oxygen uptake of the first ventilatory threshold from baseline assessment; Time, time spent performing the simulated ultramarathon; Distance, distance performed in the simulated ultramarathon; Speed, calculated average run speed of the ultramarathon based on time and distance. Mean (± standard deviation; SD) and Cohen's d for group comparisons in last row.

### 2.4 RR measurements and calculation of DFA a1

A Polar H10 (Polar Electro Oy, Kempele, Finland) HR monitoring (HRM) device with a sampling rate of 1000 Hz was used to detect RR intervals in all individuals during the Pre and Post treadmill run over 5 min. All RR data were recorded with a Suunto Memory Belt (Suunto, Vantaa, Finland), downloaded as text files, and then imported into Kubios HRV Software Version 3.4.3 (Biosignal Analysis and Medical Imaging Group, Department of Physics, University of Kuopio, Kuopio, Finland; Tarvainen et al., 2014). Kubios preprocessing settings were set to the default values including the RR detrending method which was kept at “Smoothn priors” (Lambda = 500). DFA a1 window width was set to 4 ≤ n ≤ 16 beats. The RR series was then corrected by the Kubios “automatic method” (Lipponen & Tarvainen, 2019) and relevant parameters exported as text files for further analysis. DFA a1 and average HR were calculated from the RR data series of the 2 min time window consisting of the start of minute 4 to the end of minute 5 of the treadmill exercise in both Pre and Post conditions. Two min time windowing was chosen based on previous calculations as to the minimal required beat count (Chen et al., 2002). Artifact levels measured by Kubios HRV were below 5%. This limit was previously shown to have minimal effect on DFA a1 during exercise (Rogers, Giles, Draper, Mourot et al., 2021).

### 2.5 Statistics

Statistical analyses of means and standard deviations were performed for demographic data, Pre and Post treadmill run DFA a1, average HR and VO_2_ in Microsoft Excel 365. Additional statistical analysis was performed using SPSS 23.0 (IBM Statistics, United States) for Windows (Microsoft, USA). The Shapiro–Wilk test was applied to verify the Gaussian distribution of the data. The degree of variance homogeneity was verified by the Levene's test. To account for the unbalanced and small participant numbers of the elite ultramarathon runners group comparison of demographic data, data of baseline assessment, pre intervention data and to analyze the effects of the intervention (Pre vs. Post) on dependent variables (DFA a1, HR, RE, and CMJ) were employed via effect size calculation (Coe, 2002) (the mean difference between scores divided by the pooled standard deviation of group comparison and Pre versus Post comparison of each variable). The interpretation of effect sizes is based on Cohen's thresholds for small effects (d < 0.5), moderate effects (d ≥ 0.5), and large effects (d > 0.8) (Cohen, 1988).

## 3 RESULTS

**FIGURE 1:**
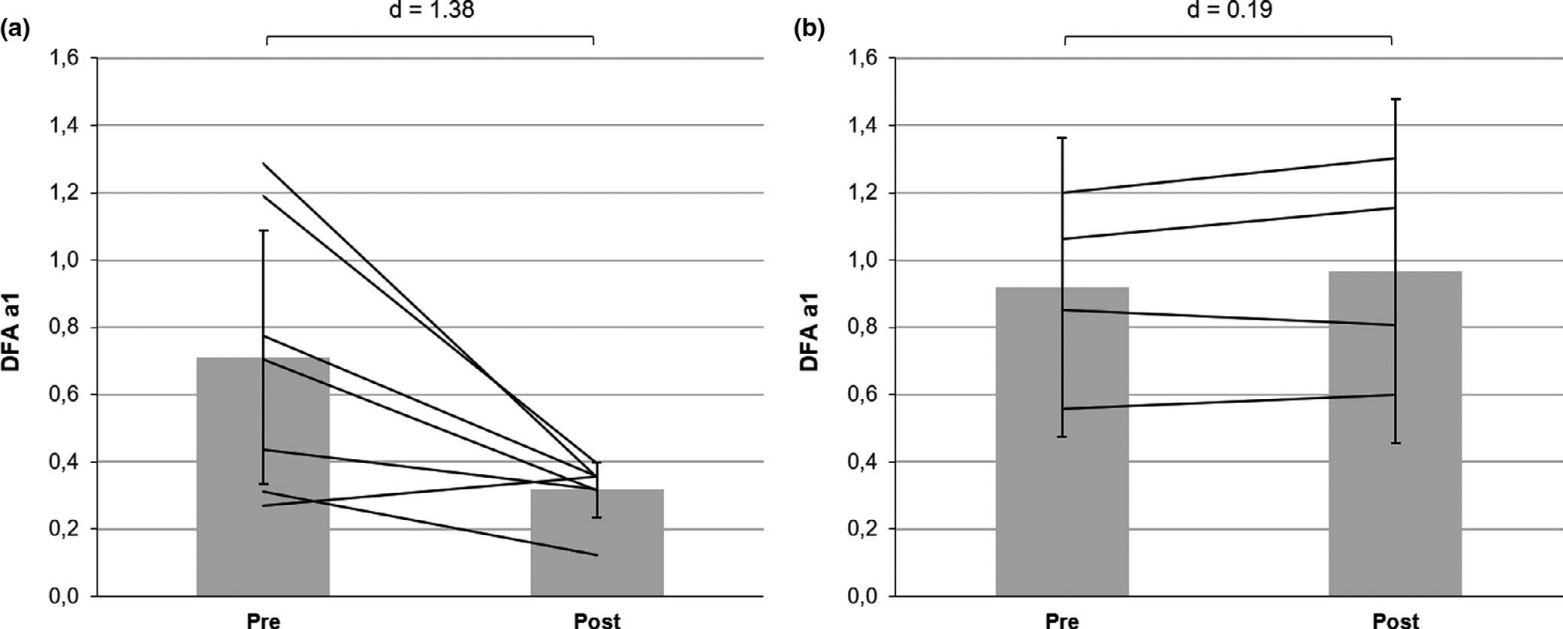
(a) Mean, 95% confidence interval and individual responses while running on a treadmill at 3 m/s for DFA a1 Pre and Post ultramarathon run (FG) in seven participants, (b) Mean, 95% confidence interval and individual responses while running on a treadmill at 3 m/s for DFA a1 Pre and Post daily activity (CG) in four participants

Mean and standard deviations for measured parameters are listed in Table 2 for each group (FG vs. CG). There were only small effect sizes in group comparison in demographic data and data from baseline assessment (Table 1). Pre intervention data showed small to medium effect sizes in comparison of both groups in dependent variables HR, DFA a1, RE, and CMJ (Table 2). In Pre versus Post comparisons, data showed a decline with large effect size in DFA a1 (d = 1.38) and CMJ vertical peak force (d = 0.82) post intervention only for FG, with minor differences and small effect sizes in HR (d = 0.02), RE (d = 0.21) or CMJ jump height (d = 0.43). CG showed only minor differences with small effect sizes in DFA a1 (d = 0.19), HR (d = 0.15), RE (d = 0.31) and CMJ vertical peak force (d = 0.02), and jump height (d = 0.09) (Figure 1).

## 4 DISCUSSION

The aim of this study was to determine if a simulated ultramarathon run‐induced changes in a nonlinear HRV index of fractal correlation properties, DFA a1, during dynamic exercise. Since the ultramarathon has been shown to cause major perturbation of many metabolic, systemic, and neuromuscular systems (Knechtle & Nikolaidis, 2018; Ramos‐Campo et al., 2016), it is ideal for investigating whether a HRV index representing overall organismic demand also exhibits analogous alterations while still performing the exercise. This particular index is especially well suited for the assessment of overall physiologic status during activity by virtue of its excellent dynamic range over mild, moderate, and severe exercise intensity domains (Gronwald et al., 2020). A major finding of this report is that after a 6 h ultramarathon, DFA a1 was markedly suppressed while running at a pace close to the aerobic threshold. Vertical peak force decreases from CMJ assessment confirmed fatigue‐induced changes in the neuromuscular function of the lower‐limbs. Despite the expected systemic effects, neither HR nor running economy appeared to be altered after the ultramarathon. Past analyses have shown variable effects on measures of running economy post ultramarathon with both higher and neutral oxygen usage at a fixed running speed (Scheer et al., 2018; Vernillo et al., 2019). In regard to HR over the course of a marathon, it appears that this metric is not very helpful in monitoring ongoing fatigue. HR can remain stable without much upward drift over the course of a marathon, at the cost of a slight decrease in speed (Billat et al., 2012). Therefore, if one were attempting to track signs of metabolic distress by observing HR, VO_2,_ or DFA a1 in this particular study, only DFA a1 would have revealed changes while activity was ongoing. As compared with Pre measurements, DFA a1 was markedly suppressed in all athletes during the exercise at a fixed low intensity pace after the ultramarathon, comprising values well past uncorrelated patterns and falling into the anticorrelated range. These values are generally associated with the highest exercise intensity domain and should not occur during low to moderate work rates (Gronwald & Hoos, 2020). In accordance with this observation, prior studies of prolonged cycling exercise (60 min or until voluntary exhaustion) with constant power at 90% to 100% of the second lactate threshold, showed DFA a1 exhibiting a clear decrease comparing the beginning and end of the exercise bout, potentially showing an effect of fatigue (Gronwald et al., 2018, 2019). In the present study, all but one of the FG individuals had suppression of DFA a1 from their Pre‐values. Although the CG did not have similar DFA a1 values compared to the FG before the ultramarathon they did not have a meaningful decline, when tested again after normal daily activity. In terms of running pace, the ultramarathon speed was well below that of the treadmill test of 3 m/s and below the AT as demonstrated by baseline VO_2_ measurements. Despite this point, it appears that blood lactate does accumulate above baseline but still remains at a steady state during an ultramarathon run (Jastrzębski et al., 2015; Ramos‐Campo et al., 2016). Therefore, it seems that blood lactate could underestimate the severity of this type of long duration exercise in terms of whole body systemic effects.

The mechanism of DFA a1 decline during both increasing exercise intensity and high organismic demand revolves around autonomic nervous system balance as well as other potential factors (Sandercock & Brodie, 2006; Papaioannou et al., 2013; White & Raven, 2014; Michael et al., 2017). As overall demand rises there is a withdrawal of the parasympathetic and stimulation of the sympathetic system (White & Raven, 2014) affecting the sinoatrial node leading to a loss of fractal correlation properties of the HR times series. This can also be described in terms of a “networking” process (Balagué et al., 2020), related to integration of many metabolic, neuromuscular and hormonal inputs. With increasing exercise intensity and/or fatigue it seems that organismic regulation starts to disengage subsystems (e.g., dissociation of cardiac and respiratory systems) in terms of a disintegration, decoupling, and segregation process (Gronwald et al., 2020). This behavior could be interpreted as a protective feedback mechanism where interactions of subsystems fail before the whole system fails. Interestingly, studies have indicated that DFA a1 rises in the immediate post ultramarathon recovery period during supine resting conditions, showing highly correlated patterns with increased correlation properties of HR time series (Martínez‐Navarro et al., 2019). This activity could be explained as a systematic reorganization of the organism with increased correlation properties in cardiac autonomic regulation with a predominance of parasympathetic activity during passive or active recovery with very low exercise intensity (parasympathetic reactivation) (Casties et al., 2006; Kannankeril & Goldberger, 2002; Stanley et al., 2013). It may also be related to a counter regulation (overcompensation) of the organism to the prior load (Hautala et al., 2001). The organism responds with a highly correlated behavior signifying more order in recovery (Balagué et al., 2020; Gronwald et al., 2019).

### 4.1 Limitations and future directions

A limitation of this study is a lack of time related detail of speed, HR, and DFA a1 during the ultramarathon. Additional study looking at a comprehensive analysis of DFA a1 and related metrics throughout the entire run would certainly be of interest, especially at what point does its behavior begin to deviate from normal. Periodic blood lactate determinations would also have been of interest, but difficult on a practical basis. Although a derived running pace can be inferred from the overall session distance/time, it is possible that some heterogeneity was present. The overall derived pace of 2 m/s was consistent with an intensity below the AT since VO_2_ measurements at 3 m/s were usually slightly above or below the AT. Two female participants were included but just one was in the FG. Given the limited data on female participants further evaluation of DFA a1 behavior during long duration endurance exercise is needed. An important potential issue in measuring DFA a1 during running may entail an artifactual suppression of correlation properties due to device bias, present in some individuals more than others (Rogers, Giles, Draper, Mourot et al., 2021). Despite possessing low artifact data, in two of the FG participants, DFA a1 was already markedly suppressed at a running speed corresponding to their VT1. For this reason, DFA a1 Pre‐values were different (with moderate effect size) in FG versus CG. Further study regarding the issue of inappropriate DFA a1 suppression at moderate running speed is needed. Sample size was relatively small but consistent with the difficulty in recruiting appropriate participants. On a practical note, the required measurement equipment consists of only a consumer grade HRM device which most athletes can easily obtain. Although this study employed a retrospective analysis to determine DFA a1, as mobile technology improves, it is conceivable that real‐time DFA a1 monitoring during endurance exercise could be used to inform an individual about current physiologic (fatigue) status and potential metabolic destabilization (Rogers and Gronwald, 2021; Gronwald et al., 2021). It is also possible that altered DFA a1 kinetics such as a delay of its decline over a given pace/distance following a training intervention could signify an improving performance status. Finally, although during race conditions, pace adjustment to mitigate DFA a1 decline is of unclear value, it certainly merits potential study during training for intensity distribution and as a safety precaution.

## 5 CONCLUSION

At the completion of an ultramarathon, DFA a1 decreased with large effect size while running at low intensity compared to pre‐race values. Despite running at a relatively easy pace, these values were consistent with those only seen at the highest levels of internal load and organismic demand. DFA a1 may offer an opportunity for real‐time tracking of physiologic status in terms of monitoring for fatigue and possibly as an early warning signal of systemic perturbation.

## CONFLICTS OF INTEREST

The authors declare that the research was conducted in the absence of any commercial or financial relationships that could be construed as a potential conflict of interest.

## AUTHOR CONTRIBUTIONS

B.R. and T.G. conceived the study. G.D. and L.M. performed the physiologic testing. B.R. wrote the first draft of the article. B.R. and T.G. performed the data analysis. All authors (B.R., G.D., L.M., and T.G.) revised it critically for important intellectual content, final approval of the version to be published, and accountability for all aspects of the work.

## INFORMED CONSENT STATEMENT

Informed consent was obtained from all subjects involved in the study.

## Data Availability

The raw data supporting the conclusions of this article will be made available by the authors, without undue reservation.
